# Handwashing sinks as reservoirs of carbapenem-resistant *Acinetobacter baumannii* in the intensive care unit: a prospective multicenter study

**DOI:** 10.3389/fpubh.2024.1468521

**Published:** 2024-10-09

**Authors:** Li Wei, Yu Feng, Ji Lin, Xia Kang, Hongdi Zhuang, Hongxia Wen, Shasha Ran, Lan Zheng, Yujing Zhang, Qian Xiang, Yan Liu, Xueqin Wu, Xiaofei Duan, Wensheng Zhang, Qu Li, Hua Guo, Chuanmin Tao, Fu Qiao

**Affiliations:** ^1^Department of Laboratory Medicine, West China Hospital, Sichuan University, Chengdu, China; ^2^Department of Infection Control, West China Hospital, Sichuan University, Chengdu, China; ^3^Center for Pathogen Research, West China Hospital, Sichuan University, Chengdu, China; ^4^Department of Infection Control, Chengdu Women and Children Hospital, Chengdu, China; ^5^Department of Infection Control, Chengdu Second People’s Hospital, Chengdu, China; ^6^Department of Infection Control, Chengdu First People’s Hospital, Chengdu, China; ^7^Department of Infection Control, Sichuan Provincial People’s Hospital, Chengdu, China; ^8^Department of Infection Control, Affiliated Hospital of Chengdu University, Chengdu, China; ^9^Department of Infection Control, The First Affiliated Hospital of Chengdu Medical College, Chengdu, China; ^10^Department of Infection Control, Chengdu Public Health Center, Chengdu, China; ^11^Department of Infection Control, Traditional Chinese Medicine Hospital of Sichuan Province, Chengdu, China; ^12^Department of Infection Control, Sichuan Provincial Maternity and Child Health Care Hospital, Chengdu, China; ^13^Department of Infection Control, Chengdu Third People’s Hospital, Chengdu, China

**Keywords:** carbapenem-resistant *Acinetobacter baumannii*, intensive care units, handwashing sinks, environmental reservoir, outbreak source

## Abstract

**Introduction:**

The extent to which sinks are contaminated by carbapenem-resistant *Acinetobacter baumannii* (CRAB) in intensive care units (ICUs) and the association between these contaminated sinks and hospital-acquired CRAB infections during the non-cluster period remains largely unknown. Here, we performed a prospective multicenter study in 16 ICUs at 11 tertiary hospitals in Chengdu, China.

**Methods:**

We sampled sinks, collected CRAB clinical isolates, and conducted whole-genome sequencing and analysis.

**Results:**

A total of 789 swabs were collected from 158 sinks, and 16 CRAB isolates were recovered from 16 sinks, resulting in a contamination rate of 10.16%. Twenty-seven clinical isolates were collected during the study period. The majority (97.67%, 42/43) of the CRAB isolates belonged to ST2, and 36 (83.72%) of them had both *bla*_OXA-23_ and *bla*_OXA-66_. The 43 strains belonged to 12 clones. One certain clone caused multiple contaminations of seven sinks in one GICU. Two clones of ST2 *bla*_OXA-23_ and *bla*_OXA-66_-carrying sink strains were likely the sources of the two clusters in the two GICUs, respectively. Five ST2 *bla*_OXA-23_-carrying isolates were found to be common clones but were recovered from two hospitals.

**Conclusion:**

The contamination rate of CRAB in handwashing sinks is high in some local ICUs, and the contaminated sinks can serve as environmental reservoirs for CRAB clusters.

## 1 Introduction

Carbapenem-resistant *Acinetobacter baumannii* (CRAB) is a common healthcare-associated infection (HAI) pathogen and has been classified as a critical priority by the World Health Organization due to the urgent need for new antibiotics to combat it (1). A systematic review conducted by Antimicrobial Resistance Collaborators showed that the CRAB contributed to 57,700 deaths globally in 2019 (2). In the intensive care unit (ICU), CRAB is particularly common and is mainly responsible for HAIs and hospital outbreaks (3–5), causing a wide range of infections, including ventilator-associated pneumonia and bloodstream infections (6–8).

The most common mechanism by which CRAB strains exert carbapenem antibiotic resistance is the production of oxacillinase (OXA)-type carbapenem-hydrolyzing class D *β*-lactamases, which are encoded by intrinsic *bla*_OXA-51-like_ genes (e.g., *bla*_OXA-51_, *bla*_OXA-64_, *bla*_OXA-65_, *bla*_OXA-66_, and *bla*_OXA-98_,) and/or acquired genes, such as *bla*_OXA-23-like_ (e.g., *bla*_OXA-23_, *bla*_OXA-27_, *bla*_OXA-49_, and *bla*_OXA-225_), *bla*_OXA-24/40-like_ (e.g., *bla*_OXA-24_, *bla*_OXA-25_, *bla*_OXA-26_, *bla*_OXA-40_, and *bla*_OXA-72_), *bla*_OXA-58-like_ (e.g., *bla*_OXA-58_ and *bla*_OXA-164_), *bla*_OXA-235-like_ (e.g., *bla*_OXA-235_ and *bla*_OXA-237_), and *bla*_OXA-143-like_ genes (9–12). The most prevalent acquired carbapenemase gene reported is *bla*_OXA-23_, followed by *bla*_OXA-24/40_ (12–16). In addition, class B metal *β*-lactamase genes (e.g., *bla*_NDM_, *bla*_VIM_, and *bla*_IMP_) have also been reported to exist in a very small number of CRAB strains (9, 10).

Handwashing sinks play an important role in infection prevention and control systems and are common in all hospitals, including in the ICU. In China, a multicenter investigation conducted in 14 provinces showed that the total installation rate of handwashing sinks increased from 69.30% in 2010 to 77.20% in 2016 in 200 hospitals (17). However, handwashing sinks can be contaminated by multi-drug-resistant organisms and cause outbreaks in hospitals (18). Previous studies have identified that certain parts of handwashing sinks, such as sink traps and sink splashing (19, 20), sink bowls and drains (21, 22), faucet aerators (23), and water taps (24), can be contaminated by CRAB during the outbreak period.

However, few studies have focused on the level of handwashing sink contamination by CRAB in different ICUs during the non-outbreak period, and the degree to which contaminated handwashing sinks are associated with hospital-acquired infections in CRAB remains largely unknown. In this study, we conducted a prospective multicenter investigation across 11 hospitals in Chengdu, with the following three objectives: (1) to evaluate the prevalence of CRAB contamination in the sinks of local ICUs during a non-outbreak period; (2) to characterize the carbapenem-resistant genes and molecular epidemiological features of CRAB strains isolated from infected patients and from handwashing sinks in these ICUs; and (3) to assess the potential role of CRAB-contaminated sinks in contributing to patient infections during the non-outbreak investigation period.

## 2 Materials and methods

### 2.1 Study setting and design

This multicenter prospective investigation was performed in 16 ICUs at 11 hospitals in Chengdu, Sichuan Province, China. The 16 ICUs included 9 general ICUs (GICUs, with a total of 279 beds and 103 handwashing sinks) and 7 neonatal ICUs (NICUs, with a total of 282 beds and 55 handwashing sinks) (Table 1). From March 2019 to August 2019, all handwashing sinks in the included ICUs were sampled once to screen for CRAB. The clinical CRAB isolates recovered from patients were collected between 2 weeks before and 3 months after the date of sampling in ICUs. If one or more sinks were positive for CRAB, the clinical isolates from this ICU or from other participating ICUs at the same hospital were recovered and transferred to West China Hospital for identification again using the Vitek II automated system (bioMérieux, Marcy l’Etoile, France). All the isolates were subjected to whole-genome sequencing (WGS) to detect homology.

**Table 1:** Characteristics of the participating ICUs and CRAB isolates from sinks and clinical samples.

Hospital	ICU no^a^	ICU beds	Sink no.	Positive sinks (%)	CRAB clinical isolate no.
Chengdu First People’s Hospital	1G	60	13	2(15.4)	16
	1 N	36	8	0(0)	0
Chengdu Second People’s Hospital	2G	22	11	7(63.6)	4
	2 N	20	4	1(25.0)	0
The First Affiliated Hospital of Chengdu Medical College	3G	28	11	4(36.4)	4
Sichuan Provincial People’s Hospital	4G	32	9	2(22.2)	3
	4 N	45	15	0(0)	0
Affiliated Hospital of Chengdu University	5G	22	10	0(0)	…
	5 N	16	4	0(0)	…
Chengdu Public Health Center	6G	16	16	0(0)	…
Chengdu Women and Children’s Hospital	7 N	80	6	0(0)	…
Sichuan Women and Children’s Hospital	8 N	80	16	0(0)	…
Sichuan Integrative Medicine Hospital	9G	25	15	0(0)	…
	9 N	5	2	0(0)	…
Chengdu Third People’s Hospital	10G	24	4	0(0)	…
West China Hospital, Sichuan University	11G	50	14	0(0)	…
Total	16	561	158	16(10.1)	27

CRAB, carbapenem-resistant *Acinetobacter baumannii*; ICU, intensive care unit.

a In this column, G indicates general ICU; N indicates neonatal ICU; no sink was positive for CRAB at the participating ICUs and not to collect CRAB clinical isolates.

The study was approved by the Ethical Committee of the West China Hospital, and the need to obtain informed consent was waived.

### 2.2 Sample sites and sampling methods

The sample points included the surfaces, faucets, bowls, drains, overflows, and drain holes of the handwashing sinks. A total of 100 cm^2^ of the sink surface and the bowl were sampled using sterile rayon swabs (Copan, Brescia, Italy) moistened with tryptic soy broth (TSB, Hopebio, Qingdao, China). A 5 × 5 cm caliper was used to confirm the sampling area. The sample points for the faucet included a surface area of 5 cm from the outer wall of the water outlet and 3–5 cm from the inner wall. For the bubbled faucets, the bubbler was removed, and the samples were sampled using sterile rayon swabs. The drain holes were sampled 3–5 cm below the drain by the swabs. All the swabs were immediately placed into 15 mL sterile tubes containing 6 mL of TSB.

### 2.3 Microbiological methods

All the tubes were incubated at 37°C overnight and centrifuged as described in our previous study (25). The supernatant was subsequently discarded, and the precipitants were resuspended in 1 mL of TSB. A 50 μL suspension was inoculated onto *Acinetobacter* selected agar plates (CHROMagar^™^, Paris, France) containing 4 μg/mL meropenem to screen for CRAB. Isolates that were pink, round, smooth, raised, and moist on the selected agar plates were suspected and identified using a matrix-assisted laser desorption/ionization–time of flight mass spectrometry (Bruker, Billerica, Massachusetts). The minimum inhibitory concentrations (MICs) of meropenem were determined using the broth microdilution method of the Clinical and Laboratory Standards Institute (CLSI) (26).

WGS was performed for all identified isolates using the Illumina HiSeq X10 platform (Illumina, San Diego, California). Subsequent reads were assembled into contigs using SPAdes version 3.14.0 (27). Precise species identification was established based on average nucleotide identity between the strains and type strains of *Acinetobacter* species using JSpeciesWS (28). The sequence of the STs was determined using the genome sequence to query the multilocus sequence typing database.^1^ Antimicrobial-resistant genes were identified using ResFinder.^2^ The draft genomes of the strains have been deposited in GenBank under BioProject number PRJNA977829. Single-nucleotide polymorphisms (SNPs) among the same ST strains were analyzed using Snippy version 4.6.0.^3^ Recombination regions were predicted using Gubbins version 2.4.1 (29) under the GTRGAMMA model with a maximum of 50 iterations. A cutoff of equal to or less than 11 high-quality SNPs was used to define clones within the same ST strains, as described in a previous study (30).

## 3 Results

### 3.1 The contamination of sinks by CRAB

In total, 789 swabs were collected from 158 handwashing sinks in 16 ICUs at 11 hospitals, and 16 CRAB isolates were recovered from 16 handwashing sinks in 5 ICUs at 5 hospitals (Table 1). The total contamination rate of CRAB in the handwashing sink was 10.16% (16/158, 95% CI 5.40–14.83%). The contamination rate in different ICUs ranged from 0 to 63.6%, and the sink contamination rate in the GICU was 15.29% (95% CI 7.75–21.38%), while it was 3.57% (95% CI 0.0–5.35%) in the NICU. Of the 16 sink CRAB isolates, 15 were recovered from the GICUs and belonged to ST2 (12 isolates carrying both *bla*_OXA-23_ and *bla*_OXA-66_ carbapenemase genes, 1 isolate harboring both *bla*_NDM-1_ and *bla*_OXA-98,_ and 2 isolates carrying only *bla*_OXA-23_). The remaining CRAB isolate was recovered from a NICU and belonged to ST203, and this isolate harbored both *bla*_OXA-23_ and *bla*_OXA-66_ (Table 2). The 16 sink isolates could be assigned to 6 clones.

**Table 2:** CRAB recovered from sinks and clinical samples.

Isolate	ICU^a^	ST	Clone^b^	Carbapenemase	Meropenem MIC, mg/L	Source
090737	1G	2	2a	OXA-23	64	Sputum
090738	1G	2	2a	OXA-23	64	Sputum
**090698**	1G	2	2b	OXA-23, OXA-66	32	**Sink**
090713	1G	2	2b	OXA-23, OXA-66	32	Sputum
090714	1G	2	2b	OXA-23, OXA-66	32	Sputum
090715	1G	2	2b	OXA-23, OXA-66	64	Sputum
090736	1G	2	2b	OXA-23, OXA-66	32	Sputum
090739	1G	2	2b	OXA-23, OXA-66	32	Sputum
090740	1G	2	2b	OXA-23, OXA-66	32	Sputum
090745	1G	2	2b	OXA-23, OXA-66	64	Sputum
**090697**	1G	2	2c	OXA-23, OXA-66	64	**Sink**
090742	1G	2	2c	OXA-23, OXA-66	64	Sputum
090747	1G	2	2c	OXA-23, OXA-66	64	Sputum
090748	1G	2	2c	OXA-23, OXA-66	64	BALF
090741	1G	2	2d	OXA-23, OXA-66	32	Sputum
090743	1G	2	2d	OXA-23, OXA-66	32	Sputum
090746	1G	2	2e	OXA-23, OXA-66	64	Sputum
090744	1G	2	2f	OXA-23, OXA-66	32	Sputum
**090685**	2G	2	2g	NDM-1, OXA-98	64	**Sink**
**090686**	2G	2	2g	OXA-23, OXA-66	64	**Sink**
**090688**	2G	2	2g	OXA-23, OXA-66	64	**Sink**
**090690**	2G	2	2g	OXA-23, OXA-66	64	**Sink**
**090693**	2G	2	2g	OXA-23, OXA-66	64	**Sink**
**090695**	2G	2	2g	OXA-23, OXA-66	64	**Sink**
**090696**	2G	2	2g	OXA-23, OXA-66	64	**Sink**
090718	2G	2	2g	OXA-23, OXA-66	64	Sputum
090767	2G	2	2g	OXA-23, OXA-66	64	CSF
090768	2G	2	2g	OXA-23, OXA-66	64	Sputum
090766	2G	2	2h	OXA-23, OXA-66	32	Sputum
**090694**	2 N	203	…	OXA-23, OXA-66	64	**Sink**
**090700**	3G	2	2i	OXA-23, OXA-66	32	**Sink**
**090701**	3G	2	2i	OXA-23, OXA-66	64	**Sink**
090717	3G	2	2i	OXA-23, OXA-66	64	Sputum
090749	3G	2	2i	OXA-23, OXA-66	64	BALF
090750	3G	2	2i	OXA-23, OXA-66	32	BALF
**090699**	3G	2	2j	OXA-23, OXA-66	32	**Sink**
**090703**	3G	2	2j	OXA-23, OXA-66	32	**Sink**
090716	3G	2	2k	OXA-23, OXA-66	64	BALF
**090704**	4G	2	2a	OXA-23	64	**Sink**
**090705**	4G	2	2a	OXA-23	64	**Sink**
090769	4G	2	2a	OXA-23	32	Sputum
090770	4G	2	2L	OXA-23, OXA-66	64	Sputum
090771	4G	2	2m	OXA-23, OXA-66	64	BALF

BALF, bronchoalveolar lavage fluid; CSF, cerebrospinal fluid; ICU, intensive care unit; MIC, minimum inhibitory concentration; ST, sequence type; CRAB, carbapenem-resistant *Acinetobacter baumannii*. Strains from sinks are highlighted in bold.

a ICU designation is available in Table 1.

b ST number and clone designation (e.g., 2a representing clone a of ST2).

### 3.2 Characteristics of clinical isolates

A total of 27 CRAB clinical isolates were recovered from 4 of the 5 ICUs with CRAB-contaminated sinks. The vast majority of the 27 CRAB clinical isolates were recovered from sputum (*n* = 21, 77.8%), whereas the remaining were from bronchoalveolar lavage fluid (BALF, *n* = 5, 18.5%) and cerebrospinal fluid (*n* = 1, 3.7%). All of the clinical isolates belonged to ST2. Twenty-four of them harbored both *bla*_OXA-23_ and *bla*_OXA-66_ carbapenemase genes, while the other three carried only *bla*_OXA-23_ (Table 2). The 27 clinical isolates could be assigned to 12 clones.

### 3.3 Diverse clonal backgrounds of CRAB isolates and sink as a source of transmission in a GICU

**Figure 1:**
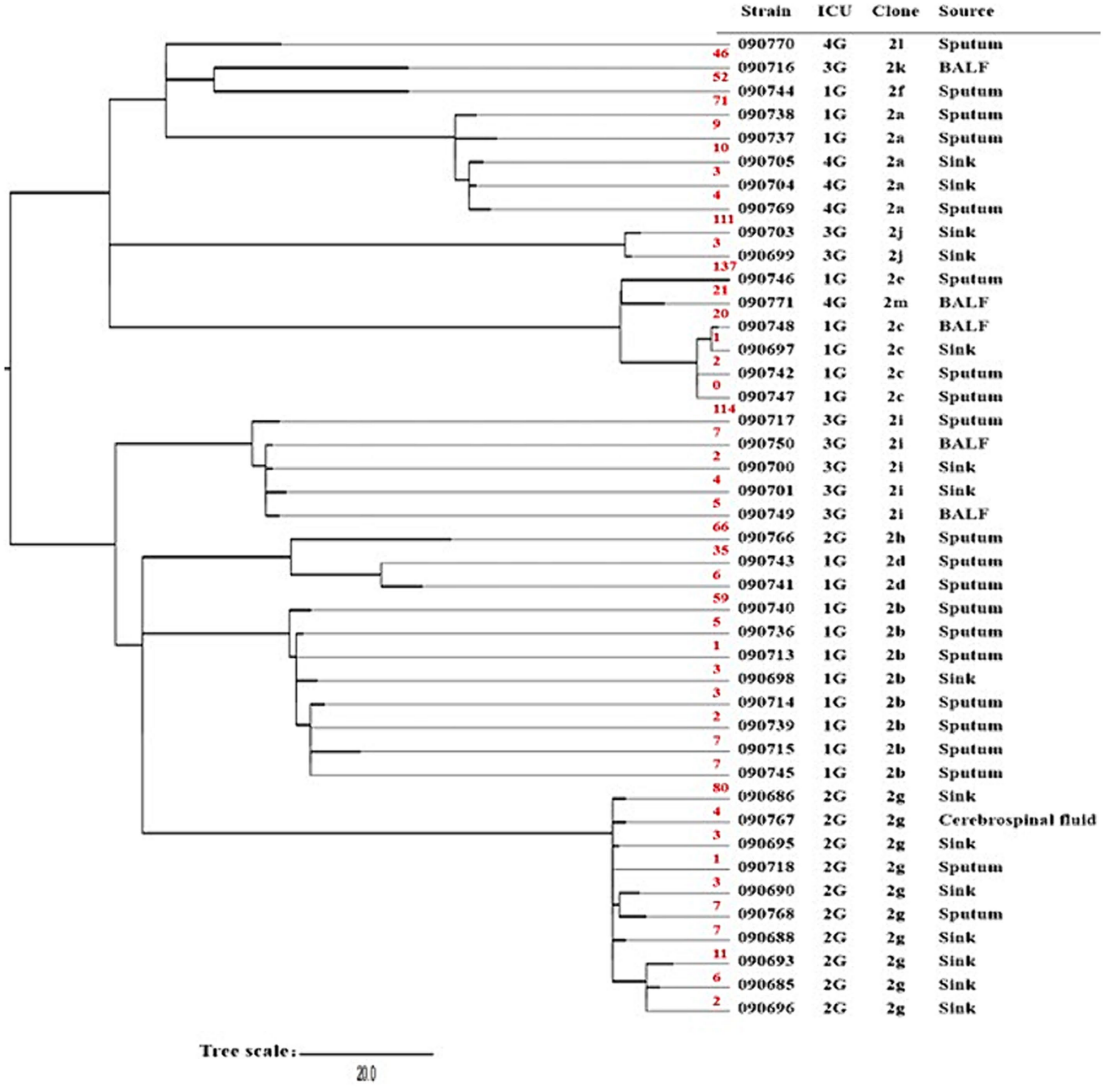
Phylogenetic trees of sequence type (ST) 2 carbapenem-resistant *Acinetobacter baumannii* strains. Shown are the strain name, the ICU where the strain was recovered (see Table 1), and the clone to which the strain belonged (see Table 2). The number of single-nucleotide polymorphisms between the adjacent two strains is indicated in red. Abbreviations: BALF, bronchoalveolar lavage fluid; ICU, intensive care unit.

A total of 18 ST2 CRAB isolates were recovered from ICU 1G, including 2 isolates from two sinks, 15 isolates from sputum, and 1 isolate from BALF. The 18 isolates could be assigned to 6 clones. Seven clinical isolates recovered from sputum, along with one sink isolate (090698), harbored both *bla*_OXA-23_ and *bla*_OXA-6_. These strains shared 1–7 SNPs, and therefore belonged to a common clone (clone 2b) (Figure 1), indicating an outbreak. Four (090713, 090714, 090715, and 090736) of these seven clinical isolates were recovered before the sink sampling date, and the other three clinical isolates (090739, 090740, and 090745) were recovered after the sink sampling date. These three isolates shared smaller SNPs (2, 4, and 2, respectively) with the clinical isolate (090713) than with the sink isolate (5, 7, and 5, respectively); therefore, the sink isolate (090698) was likely to be contaminated but not the exact route of transmission.

Another isolate (090697) from another sink shared 0–2 SNPs with three clinical isolates (two isolates were recovered from sputum, and one was recovered from BALF) and therefore belonged to a common clone (clone 2c) (Figure 1), also indicating an outbreak in the ICU 1G. All three clinical isolates were recovered at a later date than the sink sampling date; therefore, the sink was likely to be the route of infection transmission. However, the two sink isolates in this ICU shared 117 SNPs, indicating that they were assigned to two strains. The remaining six clinical isolates belonged to four clones but were not the same as the two sink isolates from ICU 1G.

### 3.4 A clone of ST2 *bla*_OXA-23_ carrying CRAB transmission in two GICUs

There were five isolates (two clinical isolates from ICU 1G, one clinical isolate, and two sink isolates from ICU 4G) that only carried the *bla*_OXA-23_ gene and shared 3–10 SNPs (clone 2a) (Figure 1), suggesting interhospital transmission. These two sink isolates (090704 and 090705) were recovered approximately 3 months prior to the clinical isolate (090769); therefore, the sinks were likely to constitute the route of patient transmission. Although the two clinical isolates (090704 and 090705) from ICU 1G were recovered approximately 1 month prior to the two sink isolates from ICU 4G, the exact route of interhospital transmission remains unclear.

### 3.5 A high contamination rate of CRAB in the NICU and GICU at a hospital

There were 8 (7 from ICU 2G and 1 from ICU 2 N) of the 15 sinks recovered CRAB isolates with a CRAB contamination rate as high as 53.3% in hospital 2. One sink isolate (090694) recovered from ICU 2 N belonged to ST203, but no clinical isolate was obtained during the period of the study in this ICU. Therefore, ST203 CRAB was not a common cause of infection among patients. Seven sink isolates (six harboring both *bla*_OXA-23_ and *bla*_OXA-66_ and one isolate harboring both *bla*_NDM-1_ and *bla*_OXA-98_) and three clinical isolates (two recovered from sputum and one recovered from cerebrospinal fluid) recovered from ICU 2G belonged to ST2, sharing 1–11 SNPs and belonging to a common clone (clone 2 g) (Figure 1). Only one of the three clinical isolates (090718) was recovered earlier date than the sink sampling date and shared 2–5 SNPs with the other two clinical isolates (090767 and 090768). Compared to the seven sink isolates, the two isolates (090767 and 090768) shared 3–7 SNPs and 6–14 SNPs, respectively. Therefore, the sink was likely to be contaminated but not the exact route of the transmission. The remaining clinical isolate (090766) from ICU 2G belonged to a distinct clone (2 h) and shared 35–142 SNPs with other isolates.

### 3.6 A cluster of ST2 *bla*_OXA-23_ and *bla*_OXA-66_-carrying CRAB in a GICU

In ICU 3G, four sinks were contaminated by CRAB, which belonged to the ST2 type and carried both *bla*_OXA-23_ and *bla*_OXA-66_ genes. Two of the four sink isolates (090700 and 090701) shared 2–7 SNPs with three clinical isolates (090717, 090749, and 090750), indicating the presence of a common clone (clone 2i). Although one (090717) of the three clinical isolates was recovered at an earlier date than the sink sampling date, the other two (090749 and 090750) clinical isolates shared fewer SNPs (3–5 and 2–4) with the two sink isolates than with the clinical isolate (8, 7). Therefore, these two contaminated sinks were likely to be the route of the transmission. The other two sink isolates (090699 and 090703) shared three SNPs and belonged to another common clone (2j) (Figure 1). The remaining clinical isolate (090716) in this ICU belonged to clone (2 k) alone, sharing 46–122 SNPs with other isolates.

## 4 Discussion

To our knowledge, this study was the first multicenter investigation on handwashing sink contamination by the CRAB in China. Unlike most studies in which the handwashing sink was sampled during an outbreak period, this study was conducted during a non-outbreak period in the included ICUs. The results showed that the contamination rate of handwashing sinks ranged from 0 to 63.6% in different ICUs, while other investigations did not find CRAB contamination in handwashing sinks during non-outbreak scenarios (31, 32). It has been well documented in the literature that the contamination level of handwashing sinks is related to the improper use, the size and depth of the sink, and the frequency of environmental cleaning (33). This study further revealed that the contamination rate of CRAB in handwashing sinks was significantly higher than that of carbapenem-resistant *klebsiella pneumoniae* (CRKP) or carbapenem-resistant *klebsiella oxytoca* (CRKO), with a rate of 10.1, 3.8, and 3.8%, respectively, during the same period, as we previously reported (25). This disparity may be attributed to the biofilm-forming capability of *Acinetobacter baumannii*, which enables it to survive on various surfaces more effectively than CRKP (34). In addition, the higher prevalence of CRAB compared to CRKP in local settings likely contributed to this difference (15).

All 27 clinical strains of CRAB obtained in our study were sourced from GICU, and this may be attributed to several factors: Patients in the GICU suffer from more severe illnesses, have longer hospital stays, experience higher rates of CRAB colonization, and are subjected to increased antibiotic usage (35, 36). Furthermore, a lack of awareness regarding the prevention and control of HAIs and insufficient cleaning and disinfection practices in GICU environments, including handwashing sinks, may contribute to CRAB infections among GICU patients (22, 37). In China, hospitals have already possessed relatively good resources for neonatal care and treatment (38), which likely results in fewer infections occurring in the NICU. It has been reported that CRAB strains carrying class B metallo-*β*-lactamase genes (e.g., *bla*_NDM_, *bla*_VIM_, and *bla*_IMP_) are prevalent in pediatric populations (16, 39). However, our findings indicate that the dominant strains identified in the GICU sinks carried class D β-lactamase genes, specifically *bla*_OXA-23_, with only one strain carrying *bla*_NDM-1_ isolated from a GICU sink.

In this study, we found that sink contamination by CRAB was not the major source of CRAB clinical infections in most ICUs in this study. However, at least three clones (clone 2c in ICU 1G, clone 2a in ICU 4G, and clone 2i in ICU 3G) of ST2 CRAB sink isolates were demonstrated to be the sources of patient infections or outbreaks. The results indicated that these three ICUs should take some proven measures to reduce the microbial contamination rate of sinks, including disinfection, use of drain covers, or even rebuilding the handwashing sink (21, 40, 41). In addition, we identified a transmission of clone 2a between two hospitals, along with two sink strains that were detected prior to a clinical strain in one of the hospitals. This finding underscores the need to pay close attention to hospital sewage contaminated with antibiotic-resistant bacteria (ARB) and antibiotic-resistant genes (ARGs) (42). Hospital sewage may serve as a potential reservoir for CRAB, highlighting the importance of strengthening disinfection, management, and monitoring protocols for hospital sewage in local settings (43).

ST2 is the most dominant type of CRAB in the world as well as in China (42). The main mechanism of carbapenem resistance in *Acinetobacter baumannii* is the acquisition of carbapenem-hydrolyzing oxacillinase-encoding genes, and *bla*_OXA23_ is by far the most widespread in most countries (44). In this study, all of the clinical isolates and all but one of the sink isolates belonged to ST2. The *bla*_OXA-23_ gene was also the most common (42/43, 97.7%) oxacillinase-encoding gene. In addition, we found that most CRAB isolates (37/43, 86.0%) carried both *bla*_OXA-23_ and *bla*_OXA-66_, similar to the findings of other studies (45–48). One ST2 CRAB strain isolated from a sink, carrying both *bla*_NDM-1_ and *bla*_OXA-51-like_ gene *bla*_OXA-98_, was not reported previously, which was alarming.

A strain of ST203 CRAB was recovered from a sink in a NICU. Although ST203 *Acinetobacter baumannii* has been found in cats in France and Japan (49, 50), this is the first report of ST203 CRAB recovered from a hospital environment and harboring both *bla*_OXA-23_ and *bla*_OXA-66_. Although there were no patients infected with this strain in our study, rigorous monitoring is needed to prevent the spread of ST203 CRAB from the sink to humans, especially neonates.

This study also has several limitations. First, the P traps of handwashing sinks were not sampled in our study, which means potential colonization by CRAB in these areas could have been missed. However, we sampled the drainage pipe 3–5 cm below the drainage tube, and the results can reflect the contamination degree of the drainage pipe to a certain extent. Second, the clinical strains were collected only within 2 weeks before and 3 months after sink sampling. There might have been additional cases of CRAB associated with sinks, which could have been missed. Third, sinks were only sampled once, and other environmental surfaces were not sampled; additional isolates from the sinks may have been missed. Despite these limitations, this study revealed the level of handwashing sink contamination by CRAB in different ICUs and the association of contaminated CRAB with hospital-acquired infections.

## 5 Conclusion

This study revealed that the contamination rate of CRAB in handwashing sinks was high in some local ICUs, and the contaminated sinks could serve as environmental reservoirs for CRAB clusters.

## Data Availability

The data presented in the study are deposited in the GenBank under BioProject repository, accession number PRJNA977829, available at https://www.ncbi.nlm.nih.gov/bioproject/?term=PRJNA977829.

## Footnotes

^1^ https://pubmlst.org/bigsdb?db=pubmlst_abaumannii_seqdef

^2^ http://genomicepidemiology.org/

^3^ https://github.Com/tseemann/snippy

## References

[1] Tacconelli E Carrara E Savoldi A Harbarth S Mendelson M Monnet DL. Discovery, research, and development of new antibiotics: the WHO priority list of antibiotic-resistant bacteria and tuberculosis. Lancet Infect Dis. (2018) 18:318–27. doi: 10.1016/S1473-3099(17)30753-3, PMID: 29276051

[2] Antimicrobial Resistance Collaborators. Global burden of bacterial antimicrobial resistance in 2019: a systematic analysis. Lancet. (2022) S0140-6736:02724. doi: 10.1016/S0140-6736(21)02724-0

[3] Bianco A Quirino A Giordano M Marano V Rizzo C Liberto MC. Control of carbapenem-resistant *Acinetobacter baumannii* outbreak in an intensive care unit of a teaching hospital in southern Italy. BMC Infect Dis. (2016) 16:747. doi: 10.1186/s12879-016-2036-7, PMID: 27955639 PMC5154034

[4] Zhao Y Hu K Zhang J Guo Y Fan X Wang Y. Outbreak of carbapenem-resistant *Acinetobacter baumannii* carrying the carbapenemase OXA-23 in ICU of the eastern Heilongjiang Province, China. BMC Infect Dis. (2019) 19:452. doi: 10.1186/s12879-019-4073-5, PMID: 31113374 PMC6530087

[5] Thoma R Seneghini M Seiffert SN Vuichard Gysin D Scanferla G Haller S. The challenge of preventing and containing outbreaks of multidrug-resistant organisms and *Candida auris* during the coronavirus disease 2019 pandemic: report of a carbapenem-resistant *Acinetobacter baumannii* outbreak and a systematic review of the literature. Antimicrob Resist Infect Control. (2022) 11:12. doi: 10.1186/s13756-022-01052-8 35063032 PMC8777447

[6] Niu T Xiao T Guo L Yu W Chen Y Zheng B. Retrospective comparative analysis of risk factors and outcomes in patients with carbapenem-resistant *Acinetobacter baumannii* bloodstream infections: cefoperazone-sulbactam associated with resistance and tigecycline increased the mortality. Infect Drug Resist. (2018) 11:2021–30. doi: 10.2147/IDR.S169432, PMID: 30464544 PMC6208797

[7] Pei Y Huang Y Pan X Yao Z Chen C Zhong A. Nomogram for predicting 90-day mortality in patients with *Acinetobacter baumannii*-caused hospital-acquired and ventilator-associated pneumonia in the respiratory intensive care unit. J Int Med Res. (2023) 51:030006052311614. doi: 10.1177/03000605231161481 PMC10028662 36935582

[8] Yu K Zeng W Xu Y Liao W Xu W Zhou T. Bloodstream infections caused by ST2 *Acinetobacter baumannii*: risk factors, antibiotic regimens, and virulence over 6 years period in China. Antimicrob Resist Infect Control. (2021) 10:16. doi: 10.1186/s13756-020-00876-6, PMID: 33461617 PMC7814448

[9] Müller C Reuter S Wille J Xanthopoulou K Stefanik D Grundmann H. A global view on carbapenem-resistant *Acinetobacter baumannii*. MBio. (2023) 14:e0226023. doi: 10.1128/mbio.02260-23, PMID: 37882512 PMC10746149

[10] Kim YJ Kim SI Kim YR Hong KW Wie SH Park YJ. Carbapenem resistant *Acinetobacter baumannii*: diversity of resistant mechanisms and risk factors for infection. Epidemiol Infect. (2011) 140:137–45. doi: 10.1017/S0950268811000744 21554783

[11] Brown S Amyes SGB. The sequences of seven class D β-lactamases isolated from carbapenem-resistant *Acinetobacter baumannii* from four continents. Clin Microbiol Infect. (2005) 11:326–9. doi: 10.1111/j.1469-0691.2005.01096.x, PMID: 15760431

[12] Bulens SN Campbell D McKay SL Vlachos N Burgin A Burroughs M. Carbapenem-resistant *Acinetobacter baumannii* complex in the United States—an epidemiological and molecular description of isolates collected through the emerging infections program, 2019. Am J Infect Control. (2024) 52:1035–42. doi: 10.1016/j.ajic.2024.04.184, PMID: 38692307 PMC11979794

[13] Wang M Ge L Chen L Komarow L Hanson B Reyes J. Clinical outcomes and bacterial characteristics of Carbapenem-resistant *Acinetobacter baumannii* among patients from different global regions. Clin Infect Dis. (2024) 78:248–58. doi: 10.1093/cid/ciad556 37738153 PMC10874260

[14] Wohlfarth E Kresken M Higgins PG Stefanik D Wille J Hafner D. The evolution of carbapenem resistance determinants and major epidemiological lineages among carbapenem-resistant *Acinetobacter baumannii* isolates in Germany, 2010-2019. Int J Antimicrob Agents. (2022) 60:106689. doi: 10.1016/j.ijantimicag.2022.106689, PMID: 36375774

[15] Luo Q Lu P Chen Y Shen P Zheng B Ji J. ESKAPE in China: epidemiology and characteristics of antibiotic resistance. Emerg Microbes Infect. (2024) 13:2317915. doi: 10.1080/22221751.2024.2317915, PMID: 38356197 PMC10896150

[16] Zhu Y Zhang X Wang Y Tao Y Shao X Li Y. Insight into carbapenem resistance and virulence of *Acinetobacter baumannii* from a children’s medical Centre in eastern China. Ann Clin Microbiol Antimicrob. (2022) 21:47. doi: 10.1186/s12941-022-00536-0, PMID: 36335338 PMC9637306

[17] Peng XE Dan-Hui XU Hou TY Wei-Guang LI Hong-Qiu MA Yang H. Current situation of hand hygiene facilities in Chinese multicenter hospitals. Chin J Infect Control. (2018) 17:753–8.

[18] Kizny Gordon AE Mathers AJ Cheong EYL Gottlieb T Kotay S Walker AS. The hospital water environment as a reservoir for Carbapenem-resistant organisms causing hospital-acquired infections—a systematic review of the literature. Clin Infect Dis. (2017) 64:1435–44. doi: 10.1093/cid/cix132, PMID: 28200000

[19] Landelle C Legrand P Lesprit P Cizeau F Ducellier D Gouot C. Protracted outbreak of multidrug-resistant *Acinetobacter baumannii* after intercontinental transfer of colonized patients. Infect Control Hosp Epidemiol. (2013) 34:119–24. doi: 10.1086/669093 23295556

[20] La Forgia C Franke J Hacek DM Thomson RB Jr Robicsek A Peterson LR. Management of a multidrug-resistant *Acinetobacter baumannii* outbreak in an intensive care unit using novel environmental disinfection: a 38-month report. Am J Infect Control. (2010) 38:259–63. doi: 10.1016/j.ajic.2009.07.012, PMID: 19900737

[21] Livingston SH Cadnum JL Gestrich S Jencson AL Donskey CJ. A novel sink drain cover prevents dispersal of microorganisms from contaminated sink drains. Infect Control Hosp Epidemiol. (2018) 39:1254–6. doi: 10.1017/ice.2018.192 30157984

[22] Carling PC. Wastewater drains: Epidemiology and interventions in 23 carbapenem-resistant organism outbreaks. Infec Control Hosp Epidemiol. (2018) 6:1–8. doi: 10.1017/ice.2018.138 29950189

[23] Lv Y Xiang Q Jin YZ Fang Y Wu YJ Zeng B. Faucet aerators as a reservoir for Carbapenem-resistant *Acinetobacter baumannii*: a healthcare-associated infection outbreak in a neurosurgical intensive care unit. Antimicrob Resist Infect Control. (2019) 8:205. doi: 10.1186/s13756-019-0635-y, PMID: 31893039 PMC6938019

[24] Hong KB Oh HS Song JS Lim JH Kang DK Son IS. Investigation and control of an outbreak of imipenem-resistant *Acinetobacter baumannii* infection in a pediatric intensive care unit. Pediatr Infect Dis J. (2012) 31:685–90. doi: 10.1097/INF.0b013e318256f3e6, PMID: 22466324

[25] Qiao F Wei L Feng Y Ran S Zheng L Zhang Y. Handwashing sink contamination and Carbapenem-resistant *Klebsiella* infection in the intensive care unit: a prospective multicenter study. Clin Infect Dis. (2020) 71:S379–85. doi: 10.1093/cid/ciaa1515, PMID: 33367578

[26] Clinical and Laboratory Standards Institute (CLSI). Performance standards for antimicrobial susceptibility testing, 29th. 29th ed. Wayne, Pennsylvania, USA: Clinical and laboratory standards institute (2019).

[27] Bankevich A Nurk S Antipov D Gurevich AA Dvorkin M Kulikov AS. SPAdes: a new genome assembly algorithm and its applications to single-cell sequencing. J Comput Biol. (2012) 19:455–77. doi: 10.1089/cmb.2012.0021, PMID: 22506599 PMC3342519

[28] Richter M Rosselló-Móra R Oliver Glöckner F Peplies J. JSpeciesWS: a web server for prokaryotic species circumscription based on pairwise genome comparison. Bioinformatics. (2016) 32:929–31. doi: 10.1093/bioinformatics/btv681, PMID: 26576653 PMC5939971

[29] Croucher NJ Page AJ Connor TR Delaney AJ Keane JA Bentley SD. Rapid phylogenetic analysis of large samples of recombinant bacterial whole genome sequences using Gubbins. Nucleic Acids Res. (2015) 43:e15. doi: 10.1093/nar/gku1196 25414349 PMC4330336

[30] Ng DHL Marimuthu K Lee JJ Khong WX Ng OT Zhang W. Environmental colonization and onward clonal transmission of carbapenem-resistant *Acinetobacter baumannii* (CRAB) in a medical intensive care unit: the case for environmental hygiene. Antimicrob Resist Infect Control. (2018) 7:51. doi: 10.1186/s13756-018-0343-z, PMID: 29644052 PMC5891964

[31] Franco LC Tanner W Ganim C Davy T Edwards J Donlan R. A microbiological survey of handwashing sinks in the hospital built environment reveals differences in patient room and healthcare personnel sinks. Sci Rep. (2020) 10:8234. doi: 10.1038/s41598-020-65052-7 32427892 PMC7237474

[32] Valentin AS Santos SD Goube F Gimenes R Decalonne M Mereghetti L. A prospective multicenter surveillance study to investigate the risk associated with contaminated sinks in the intensive care unit. Clin Microbiol Infect. (2021) 27:1347.e9–1347.e14. doi: 10.1016/j.cmi.2021.02.018, PMID: 33640576

[33] Lewis SS Smith BA Sickbert-Bennett EE Weber DJ. Water as a source for colonization and infection with multidrug-resistant pathogens: focus on sinks. Infect Control Hosp Epidemiol. (2018) 39:1463–6. doi: 10.1017/ice.2018.273, PMID: 30526717

[34] Greene C Wu J Rickard AH Xi C. Evaluation of the ability of *Acinetobacter baumannii* to form biofilms on six different biomedical relevant surfaces. Lett Appl Microbiol. (2016) 63:233–9. doi: 10.1111/lam.12627, PMID: 27479925 PMC7057210

[35] Sheng WH Liao CH Lauderdale TL Ko WC Chen YS Liu JW. A multicenter study of risk factors and outcome of hospitalized patients with infections due to carbapenem-resistant *Acinetobacter baumannii*. Int J Infect Dis. (2010) 14:e764–9. doi: 10.1016/j.ijid.2010.02.2254, PMID: 20646946

[36] Playford EG Craig JC Iredell JR. Carbapenem-resistant *Acinetobacter baumannii* in intensive care unit patients: risk factors for acquisition, infection and their consequences. J Hosp Infect. (2007) 65:204–11. doi: 10.1016/j.jhin.2006.11.010 17254667

[37] Lerner AO Abu-Hanna J Carmeli Y Schechner V. Environmental contamination by carbapenem-resistant *Acinetobacter baumannii*: the effects of room type and cleaning methods. Infect Control Hosp Epidemiol. (2019) 41:1–6. doi: 10.1017/ice.2019.307 31722777

[38] Li Q Li X Zhang Q Zhang Y Liu L Cheng X. A Cross-sectional Nationwide study on accessibility and availability of neonatal care resources in hospitals of China: current situation, mortality and regional differences. Lancet Reg Health Western Pacific. (2021) 14:100212. doi: 10.1016/j.lanwpc.2021.100212 34528000 PMC8358159

[39] Chávez Rodríguez M Mascareñas De Los Santos AH Vaquera Aparicio DN Aguayo Samaniego R García Pérez R Siller-Rodríguez D. Molecular epidemiology of carbapenemase encoding genes in *A. baumannii-calcoaceticus* complex infections in children: a systematic review. JAC Antimicrob Resist. (2024) 6:dlae098. doi: 10.1093/jacamr/dlae098, PMID: 39005591 PMC11242458

[40] Gbaguidi-Haore H Varin A Cholley P Thouverez M Hocquet D Bertrand X. A bundle of measures to control an outbreak of *Pseudomonas aeruginosa* associated with P-trap contamination. Infect Control Hosp Epidemiol. (2018) 39:164–9. doi: 10.1017/ice.2017.304, PMID: 29417923

[41] Smolders D Hendriks B Rogiers P Mul M Gordts B. Acetic acid as a decontamination method for ICU sink drains colonized by carbapenemase-producing *Enterobacteriaceae* and its effect on CPE infections. J Hosp Infect. (2019) 102:82–8. doi: 10.1016/j.jhin.2018.12.009, PMID: 30579969

[42] Barancheshme F Munir M. Strategies to combat antibiotic resistance in the wastewater treatment plants. Front Microbiol. (2018) 8:2603. doi: 10.3389/fmicb.2017.02603 29387043 PMC5776126

[43] Gu D Wu Y Chen K Zhang Y Ju X Yan Z. Recovery and genetic characterization of clinically-relevant ST2 carbapenem-resistant *Acinetobacter baumannii* isolates from untreated hospital sewage in Zhejiang Province, China. Sci Total Environ. (2024) 916:170058. doi: 10.1016/j.scitotenv.2024.170058 38218490

[44] Hamidian M Nigro SJ. Emergence, molecular mechanisms and global spread of carbapenem-resistant *Acinetobacter baumannii*. Microb Genom. (2019) 5:e000306. doi: 10.1099/mgen.0.000306 31599224 PMC6861865

[45] Chen Y Gao J Zhang H Ying C. Spread of the *bla*_OXA-23_-containing Tn2008 in Carbapenem-resistant *Acinetobacter baumannii* isolates grouped in CC92 from China. Front Microbiol. (2017) 8:163. doi: 10.3389/fmicb.2017.00163 28220115 PMC5292404

[46] Al-Hassan L Elbadawi H Osman E Ali S Elhag K Cantillon D. Molecular epidemiology of Carbapenem-resistant *Acinetobacter baumannii* from Khartoum state, Sudan. Front Microbiol. (2021) 12:628736. doi: 10.3389/fmicb.2021.628736 33717019 PMC7952628

[47] Zhang X Li F Awan F Jiang H Zeng Z Lv W. Molecular epidemiology and clone transmission of Carbapenem-resistant *Acinetobacter baumannii* in ICU rooms. Front Cell Infect Microbiol. (2021) 11:633817. doi: 10.3389/fcimb.2021.633817, PMID: 33718283 PMC7952536

[48] Hu H Lou Y Feng H Tao J Shi W Ni S. Molecular characterization of Carbapenem-resistant *Acinetobacter baumannii* isolates among intensive care unit patients and environment. Infect Drug Resist. (2022) 15:1821–9. doi: 10.2147/IDR.S349895, PMID: 35444432 PMC9013810

[49] Kimura Y Harada K Shimizu T Sato T Kajino A Usui M. Species distribution, virulence factors, and antimicrobial resistance of *Acinetobacter spp*. isolates from dogs and cats: a preliminary study. Microbiol Immunol. (2018) 62:462–6. doi: 10.1111/1348-0421.12601 29752821

[50] Pailhoriès H Belmonte O Kempf M Lemarié C Cuziat J Quinqueneau C. Diversity of *Acinetobacter baumannii* strains isolated in humans, companion animals, and the environment in Reunion Island: an exploratory study. Int J Infect Dis. (2015) 37:64–9. doi: 10.1016/j.ijid.2015.05.012, PMID: 26093214

